# Lentiviral-Mediated Overexpression of the 18 kDa Translocator Protein (TSPO) in the Hippocampal Dentate Gyrus Ameliorates LPS-Induced Cognitive Impairment in Mice

**DOI:** 10.3389/fphar.2016.00384

**Published:** 2016-10-18

**Authors:** Wei Wang, Liming Zhang, Xiaoying Zhang, Rui Xue, Lei Li, Weixing Zhao, Qiang Fu, Weidong Mi, Yunfeng Li

**Affiliations:** ^1^Anesthesia and Operation Center, Chinese PLA General HospitalBeijing, China; ^2^Department of New Drug Evaluation, Beijing Institute of Pharmacology and ToxicologyBeijing, China; ^3^Department of Anesthesiology, The General Hospital of the PLA Rocket ForceBeijing, China; ^4^Department of Postgraduates, Hebei North UniversityZhangjiakou, China

**Keywords:** neuroinflammation, TSPO, cognitive impairment, allopregnanolone, neurogenesis, hippocampus

## Abstract

The 18 kDa translocator protein (TSPO) is involved in the immune/inflammatory response. However, the exact role that TSPO plays in neuroinflammation-induced cognitive impairment is still elusive. The purpose of our present study was to investigate the effects of lentiviral-mediated hippocampal overexpression of the TSPO in a mouse model of LPS-induced cognitive impairment. We established a mouse cognitive impairment model using systematic daily administration of lipopolysaccharide (LPS) (0.5 mg/kg). Microinjection of the dentate gyrus of the mouse with lentiviral vectors, which contained a cDNA targeting TSPO (Lv-*TSPO*), resulted in a significant increase in TSPO expression and allopregnanolone production. Mice treated with LPS showed cognitive deficits in the novel object recognition test and the Morris water maze test that could be ameliorated by TSPO overexpression. In addition, TSPO overexpression reversed LPS-induced microglial activation and accumulation of pro-inflammatory cytokines, including IL-1β, IL-6, and TNF-α. Moreover, TSPO overexpression attenuated the LPS-induced impairment of hippocampal neurogenesis. Our results suggest that local overexpression of TSPO in the hippocampal dentate gyrus alleviated LPS-induced cognitive deficits, and its effects might be mediated by the attenuation of inflammatory cytokines, inhibition of microglial activation, and promotion of neurogenesis.

## Introduction

Cognitive impairment, a common and serious symptom, deeply influences people’s quality of life worldwide. Neuroinflammation plays pivotal roles in the neuropathogenesis of diseases with cognitive impairment, such as Alzheimer’s disease (AD; Agostinho et al., 2010) and postoperative cognitive dysfunction (POCD; Wan et al., 2007). In addition, drugs such as non-steroidal anti-inflammatory drugs (NSAIDs) and proliferator-activated receptor gamma (PPARγ) agonists suppress neuroinflammation and alleviate cognitive impairment in several animal models (Steinman, 2010; Mandrekar-Colucci and Landreth, 2011; Shadfar et al., 2015). However, in clinicaltrials, there have been disappointing outcomes when these drugs were tested in patients with cognitive deficits (Breitner et al., 2011; Geldmacher et al., 2011).

Allopregnanolone, one of the most important neurosteroids, has been reported to attenuate neuroinflammation in several animal models (Wang et al., 2010; Nezhadi et al., 2016). Pre-clinical studies have demonstrated that decreased allopregnanolone levels in the brain were associated with neuroinflammatory cognitive deficits in rats (George et al., 2010). Additionally, allopregnanolone administration induced neuroprotective effects and attenuated neuroinflammatory cognitive impairment in rodents (Wang et al., 2010; Nezhadi et al., 2016). Interestingly, accumulating evidence has revealed that the 18 kDa translocator protein (TSPO), which represents the starting point and an important rate-limiting step in neurosteroidogenesis, plays an important role during neuroinflammation (Chen and Guilarte, 2008). It was reported that the level of TSPO was increased during the neuroinflammatory process, while TSPO ligands exerted anti-inflammatory and neuroprotective effects *in vivo* and *in vitro* by increasing the production of allopregnanolone. For example, TSPO ligands decreased the expression of pro-inflammatory cytokines in BV-2 cells exposed to lipopolysaccharide (LPS) (Karlstetter et al., 2014), dampened pro-inflammatory microglial reactivity in the retina (Scholz et al., 2015), attenuated pathology in a mouse model of Alzheimer’s disease (Barron et al., 2013), and even reversed the cognitive impairment induced by LPS in mice (Ma et al., 2016). Thus far, TSPO ligands have been developed as potential molecular markers to detect neuroinflammation and have been applied in the assessment of neuroinflammation in patients with several neuropathological conditions that are accompanied by cognitive impairment (Batarseh and Papadopoulos, 2010; Colasanti et al., 2014; Rissanen et al., 2014; Suridjan et al., 2015; Zurcher et al., 2015; Wang et al., 2016). These interesting data gave reason to hypothesize that downregulation of TSPO in the brain could be a risk factor for the etiology of neuroinflammatory cognitive impairment, and increased TSPO might be a novel pharmacological treatment strategy for cognitive impairment.

Although TSPO ligands exert anti-inflammatory and neuroprotective effects *in vivo*, as described above, we cannot exclude the systemic effects of TSPO ligands during neuroinflammation because TSPO is expressed at a high level in the testis, adrenal cortical, ovarian granulose, and luteal cells, as well as in the placenta brain glial cells (Papadopoulos et al., 2006). The hippocampus, an important area mediating the learning and memory process, has been implicated in the pathophysiology of many diseases associated with cognitive impairment (Biessels and Reagan, 2015; Dietrich et al., 2015). On the other hand, hippocampal neurogenesis, which can be suppressed by neuroinflammation, is closely related to cognitive performance (Chesnokova et al., 2016). Studies have also revealed that hippocampal TSPO expression is increased in systemic lupus erythematosus patients with neuroinflammatory cognitive impairment (Wang et al., 2016). However, the exact molecular and cellular mechanism of hippocampal TSPO in neuroinflammation remains unclear.

Based on the aforementioned factors, in the present study, we used a lentivirus to overexpress the TSPO protein in the hippocampal dentate gyrus. We first investigated whether overexpression of TSPO could reverse the cognitive impairment induced by LPS. Then, we investigated pro-inflammatory cytokines and microglial activation of the hippocampus. Furthermore, we examined hippocampal neurogenesis after hippocampal TSPO overexpression. The results obtained in this study provided new insights into the potential mechanisms and targets for the treatment of inflammatory cognitive impairment.

## Materials and Methods

### Animals

Male ICR mice (22 to 26 g) were obtained from the Beijing SPF Animal Technology Company (Beijing, China). All animals were housed in a temperature-controlled animal facility with a 12-h light-dark cycle (lights on at 6:00 AM) and had full access to water and food *ad libitum*. Animal experiments were performed in compliance with the current laws of China and the National Institutes of Health Guide for the Care and Use of Laboratory Animals (NIH publication No. 86–23, revised 1996).

### Plasmid Construction

Recombinant pGC-LV-GV287-EGFP vectors with the TSPO (NM_009775) gene (LV*-TSPO*) or with a scrambled control sequence (negative control, NC) were constructed by the Genechem Company (Genechem Co. Ltd, Shanghai, China). All the viral vectors contained EGFP as a marker to track lentivirus-mediated target gene expression by fluorescence microscopy.

### Preparation of Lentiviral Stocks

The viral particles were produced by transient transfection of HEK 293T cells with a transfer plasmid. In brief, the pGC-LV-GV287-EGFP-TSPO/NC vectors, mixed with pHelper1.0 and pHelper2.0, were co-transfected into HEK-293T cells with LipofectamineTM 2000 (Invitrogen, Shanghai, China) according to the manufacturer’s instructions. Forty-eight hours after transfection, the viral supernatants were collected, filtered through 0.45 μm polyvinylidene fluoride membranes, and then centrifuged. To ensure comparability among different virus preparations, the virus concentration was determined by real-time RT-PCR to measure the number of integrated copies of 293T cells and was reported as transducing units (2 × 10^8^ tu/ml). After transduction, the vectors were re-suspended in 10% sucrose/PBS and stored at -80°C.

### Experimental Design

**FIGURE 1:**
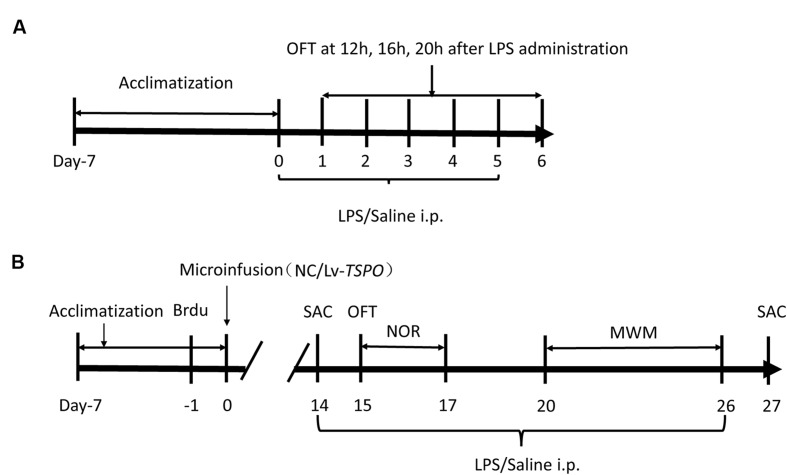
**Experimental design. (A)** Treatment schedule and test order for the optimized dose of lipopolysaccharide (LPS). LPS or saline was administered consecutively for 6 days following a 1-week acclimatization. The open field test (OFT) was performed 12, 16, 20, and 24 h after LPS/saline administration over the subsequent 6 days. **(B)** Treatment schedule and test order for evaluating the effects of TSPO overexpression and LPS. Brdu was injected three times at 3-h intervals. The next day, lentiviral vectors containing TSPO-cDNA (Lv-*TSPO*) or the negative control (NC) sequence were infused into the bilateral dentate gyrus (2 × 10^8^ tu, 1 μl/side) of the mouse hippocampus. Behavioral tests were carried out from day 15 to day 26. LPS (0.5 mg/kg) or saline was injected (*i.p.*) once daily from day 14 to day 26, and the animals were sacrificed on day 27. MWM, Morris water maze; OFT, open field test; NOR, novel object recognition; LV, lentiviral vectors; SAC, sacrifice.

Bromodeoxyuridine (Brdu) and LPS (*Escherichia coli* EH100) were purchased from Sigma–Aldrich (St. Louis, MO, USA). Both were dissolved in saline and administered by intraperitoneal injection (*i.p.*) in a volume of 10 ml/kg. First, we explored the optimal dose of LPS (0.2, 0.5, and 1 mg/kg, *i.p.)* that did not affect locomotor activity following consecutive administrations for 6 days (**Figure 1A**). The open field test (OFT) was conducted 12, 16, 20, and 24 h after each injection (days 1–6).

In the next phase, the mice were divided into four groups: negative control (NC) + saline; NC + LPS; Lv-*TSPO* + saline; Lv-*TSPO* + LPS. One day before virus injection, Brdu (100 mg/kg) was injected (*i.p.*) three times at 3 h intervals. On day 0, the Lv-*TSPO* or NC virus was microinjected into the bilateral dorsal dentate gyrus. Briefly, mice were anesthetized with chloral hydrate (400 mg/kg, Sinopharm Chemical Reagent Co., Ltd, Shanghai, China) before they were placed in a stereotaxic holder (Kopf Instruments, Tujunga, CA, USA). A 33-gage Hamilton microsyringe was inserted into the dorsal dentate gyrus (AP, -1.7 mm from the bregma; ML, ±1.8 mm from the midline; DV, -2.0 mm from the dura) on each side. Lentiviral vectors (1 μl/side) containing cDNA targeting TSPO or the NC were infused at a rate of 0.2 μl/min using a UMP3 microsyringe injector and a Micro4 controller (World Precision Instruments, Sarasota, FL, USA). The needle was slowly retracted after an additional 5 min to assure adequate diffusion of the vectors. From day 14 to day 26, LPS or saline was administered daily by intraperitoneal injection. Behavior tests were conducted 20 to 24 h after each injection of LPS or saline (**Figure 1B**).

### Behavioral Experiments

#### Open Field Test (OFT)

Mice were placed in the corner of a plastic box (60 cm × 60 cm × 23 cm) in which the base was divided into 16 equal sectors; after a 5-min acclimation period, the number of crossings (with all four paws placed into a new square) and rearings (raising the forepaws) were recorded over the next 5 min. The open field box was cleaned with 5% ethyl alcohol to hide animal clues.

#### Novel Object Recognition (NOR) Test

The NOR test was carried out in a dimly lit room. During the habituation session, the mice were allowed to become familiar with the testing box for 5 min on day 15. Twenty-four hours later, the mice were placed in the same apparatus and were individually exposed to two identical objects placed in two corners of the box for 5 min (the sample session). After another 24 h interval (on day 17), the mice were returned to the box with a previously presented familiar object and a novel one for 5 min (the test session). Exploration was defined as initiatively facing, sniffing or touching the object (within 2 cm from the object). The accumulative time exploring each object (Tf and Tn for familiar and novel objects, respectively) was recorded to determine the recognition index [RI = Tn/(Tn + Tf)].

#### Morris Water Maze (MWM) Test

The MWM test was performed as described previously with minor modifications (Vorhees and Williams, 2006). Behavioral testing was conducted in a circular pool (122 cm diameter, 35 cm depth), which was divided into four equally spaced quadrants. The pool was filled to a depth of 17 cm with water (21 ± 1°C) made opaque by the addition of powdered milk. A circular platform (10 cm^2^) was placed in the center of one of the quadrants 1.5 cm below the surface of the water. The pool was located in a well-lit room with some external cues, which remained in the same location throughout the acquisition and the probe trials. On day 20, the mice were individually placed in the pool facing the wall four times (1 min each time and each time starting in a different quadrant) without the platform to become accustomed to the pool. From day 21 to day 25, the mice were individually placed in the pool at different starting points, except for the target quadrant containing the hidden platform. The animals were trained to escape by swimming and climbing onto the platform during the acquisition trials (4 trials × 5 days, 20 trials in total). The mice that failed to find the hidden platform within 60 s were guided toward it and stayed there for 10 s before being removed. The latency to escape by swimming onto the platform was recorded. Twenty-four hours after the last acquisition training trial, the probe trial was conducted in the absence of the platform. The number of entries and the time spent in the target quadrant were recorded, with a cut-off time of 60 s.

#### Enzyme-Linked Immunosorbent Assay (ELISA)

Concentrations of IL-1β (Biosource, Invitrogen, USA), TNF-α (Biosource, Invitrogen, USA), IL-6 (Biosource, Invitrogen, USA) and allopregnanolone (Arbor Assays, Ann Arbor, MI, USA) were examined by ELISA following the manufacturers’ instructions. Hippocampal tissues of 3 mm in diameter around the injection site were punched out. Then, the tissues were homogenized in RIPA lysis buffer (Applygen, China) on ice. Supernatant protein concentrations were determined with a BCA protein assay kit (Pierce, USA) after centrifugation at 12,000 *g* for 15 min. The absorbance was read on a spectrophotometer at a wavelength of 450 nm and a reference wavelength of 650 nm.

### Western Blot Analysis

The punched hippocampal tissues (3 mm in diameter around the injection sites on both sides) were extracted by RIPA lysis buffer (Applygen, China) plus protease inhibitor and phosphatase inhibitor cocktail (Thermo Pierce, Rockford, IL, USA). Equal amounts (50 μg) of protein were resolved by electrophoresis, transferred onto polyvinylidene difluoride (PVDF) membranes and blocked with 5% skim milk solution. The membranes were incubated with rabbit anti-TSPO (at a dilution of 1:1000; Abcam, Cambridge, MA), or β-actin (1:3000; Santa Cruz Biotechnology, Santa Cruz, CA, USA) at 4°C overnight. After washing and incubating with secondary antibodies (1:3000; Santa Cruz Biotechnology, Santa Cruz, CA, USA), the specific bands were detected and quantified using Gel-Pro Analyzer software, Version 3.1 (Media Cybernetics, Bethesda, MD, USA).

### Immunohistochemistry

Immunohistochemistry was performed as described in our previous report (Li et al., 2009) and as described by Zhang et al. (2015). In brief, free-floating brain sections were incubated for 1 day in cold PBS-plus containing rat anti-Brdu (1:200; Abcam, Cambridge, MA, USA) and mouse anti-NeuN (1:1000; Chemicon, Temecula, CA, USA), or rabbit anti-Iba1 (1:200; Jackson, MS, USA). After rinsing with PBS, the sections were incubated with Red-X-conjugated goat anti-rat IgG and FITC-conjugated goat anti-mouse IgG, or FITC-conjugated anti-rabbit IgG (both at a dilution of 1:200; Jackson, MS, USA) in PBS for 2 h. After rinsing, the sections were then mounted with Vectashield medium for fluorescence imaging (Vector Laboratories, Burlingame, CA, USA). Fluorescence analyses were performed using confocal laser microscopy.

To determine the percentage of newborn neurons among the Brdu-labeled cells, at least fifty Brdu-positive cells in the dentate gyrus were randomly identified in each animal, for which the number of cells in each category was determined. Brdu-positive cells were counted using a modified stereology protocol (Li et al., 2009). For Iba1, a marker of activated microglia, we followed Zhang’s methods (Zhang et al., 2015). Every sixth section throughout the entire hippocampus was processed for Brdu/NeuN or Iba1 immunohistochemistry. All Brdu-labeled and Iba1-labeled cells in the granular cell layer and hilus were counted blindly through a 60× objective to distinguish individual cells. The number of counted cells was multiplied by six and was recorded as the total number of labeled cells in the dentate gyrus. Images were captured using a Leica TCS SP5 confocal imaging system. Integral optical density (IOD) for Iba1 staining was quantified using Image-Pro Plus 6.0 software.

### Statistical Analysis

All data were analyzed by an observer who was blind to the experimental protocol. The data are expressed as the means ± standard error (SEM) and analyzed with the statistical analysis software GraphPad Prism, Version 6.0 (GraphPad, San Diego, CA, USA). Two-group comparisons were analyzed by a two-tailed Student’s *t*-test. Comparisons between multiple groups were carried out using two-way ANOVA with Bonferroni’s multiple comparisons *post hoc* test where appropriate. For acquisition training and spatial memory testing in the MWM task, the data were analyzed using two-way ANOVA (treatment × trial time) with repeated measures (trial days). *P* < 0.05 was considered statistically significant.

## Results

### Optimal Dose of LPS and Administration Time

**FIGURE 2:**
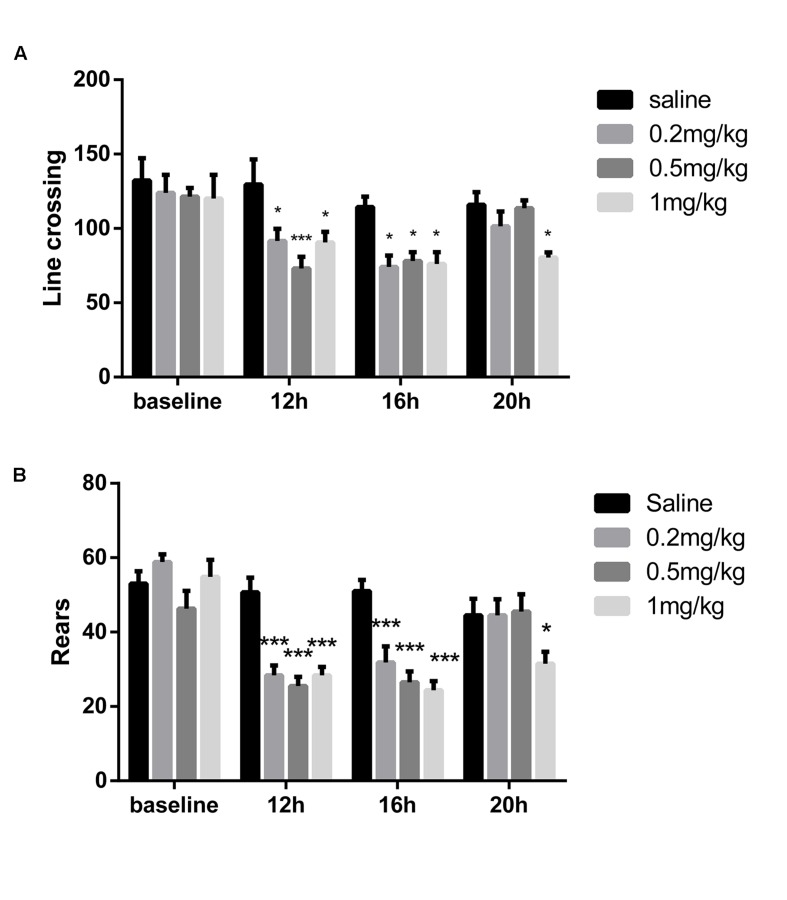
**Locomotor activity after administration of different doses of LPS.** The open field test was conducted at different times after the first LPS injection. **(A)** Line crossing times in the OFT. **(B)** Rears in the OFT. LPS (0.2, 0.5, and 1 mg/kg) decreased locomotor activity 12 and 16 h after injection, but it recovered after 20 h, except in the 1 mg/kg mice (*n* = 6). ^∗^*P* < 0.05, ^∗∗∗^*P* < 0.001 vs. saline group.

To optimize the dose of LPS without alteration of locomotor activity, three different doses of LPS were chosen for 6 days of daily intraperitoneal injection. LPS (0.2, 0.5 and 1 mg/kg) significantly decreased line crossing and rears 12 h and 16 after intraperitoneal injection in the OFT (*P* < 0.05, *n* = 6; **Figures 2A,B**). After 20 h, the mice that received 0.2 or 0.5 mg/kg LPS showed no difference in line crossing or rears compared with control mice. Only mice that received 1 mg/kg LPS exhibited decreased locomotor activity compared with control mice (*P* < 0.05). In the following 5-day tests, the mice showed results similar to those of the 1st day (data not shown). To induce significant neuroinflammation without affecting the locomotor activity, we chose 0.5 mg/kg LPS in the subsequent tests.

### Lv-*TSPO* Increased TSPO Expression and Allopregnanolone Level in the Hippocampus

**FIGURE 3:**
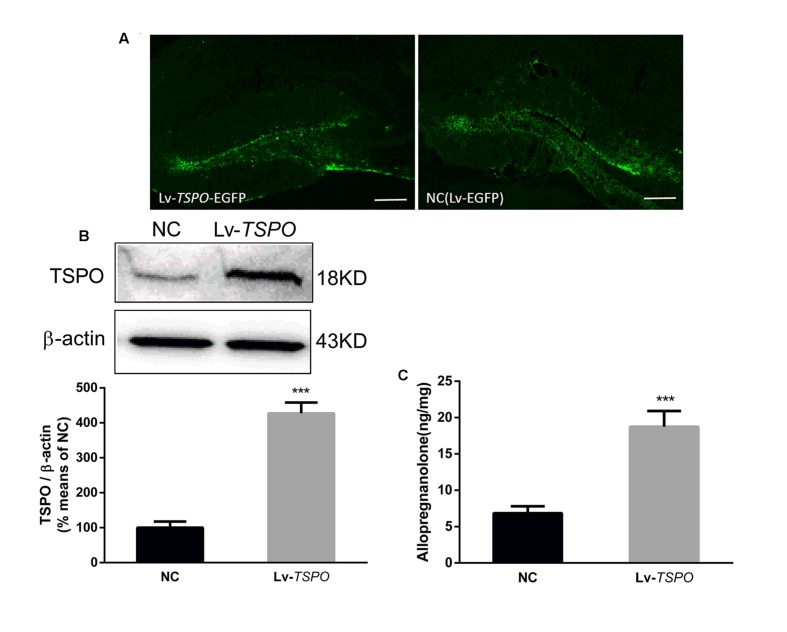
**Lv-*TSPO* increased TSPO expression and allopregnanolone levels in the hippocampus. (A)** Microinjection of Lv-*TSPO* induced high, specific expression of EGFP (green) in the dentate gyrus, as observed under fluorescence microscopy. Scale bar = 200 μm. **(B)** Lv-*TSPO* increased hippocampal TSPO expression significantly. Protein bands on the gel, and the expression level was normalized to β-actin as an internal control. Values are expressed as fold-change over the mean values of the negative control mice. **(C)** Lv-*TSPO* significantly increased allopregnanolone expression. The data are expressed as the means ± SEM (*n* = 6). ^∗∗∗^*P* < 0.001 vs. NC.

The experiment was designed for determining the effect of overexpression of TSPO mediated by lentivirus. Fourteen days after microinjection, the mice were sacrificed for analysis. We traced high and specific expression of EGFP, which was encoded in the sequence of the lentivirus, in the dentate gyrus (**Figure 3A**). Lv-*TSPO* increased TSPO expression (3 mm in diameter around the injection site on both sides) approximately fourfold compared with that in NC mice (*P* < 0.001; **Figure 3B**). The allopregnanolone level in the dentate gyrus was significantly increased after TSPO overexpression (*P* < 0.05, **Figure 3C**). Collectively, our results suggest that Lv-*TSPO* mediated the successful overexpression of TSPO in the dentate gyrus, which elevated the allopregnanolone level significantly.

### Lv-*TSPO* Ameliorates Cognitive Impairment Induced by LPS

**FIGURE 4:**
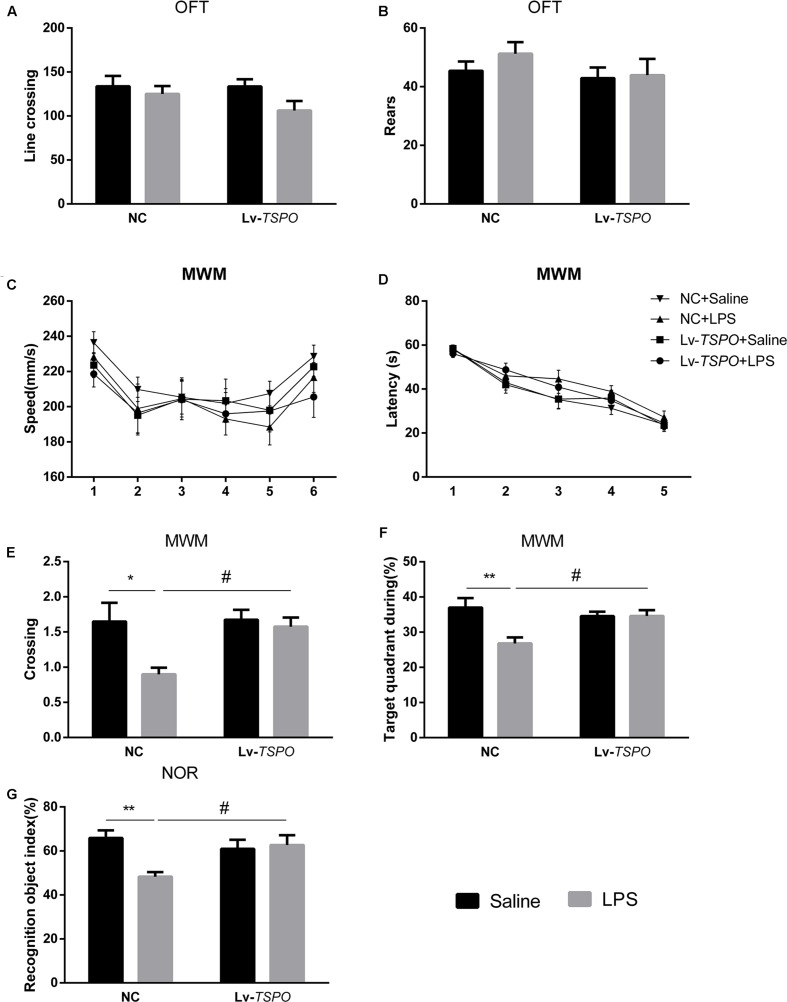
**Lv-*TSPO* ameliorates cognitive impairment in LPS-exposed mice.** All behavioral tests were performed as described in the Methods section. Both LPS and Lv-*TSPO* have no effect on spontaneous locomotor activity, as tested by the open field test. **(A)** Line crossing times in the OFT. **(B)** Rears in the OFT. **(C)** Swimming speed showed no difference among groups during the MWM test. **(D)** Escape latency during the acquisition training process showed no difference at each time point among all the groups. **(E,F)** LPS decreased the time for platform crossing and the percentage of target quadrant occupancy during time in the probe trial. The reduction was reversed by Lv-*TSPO*. **(G)** LPS decreased the index of recognition in the NOR test, while Lv-*TSPO* could reverse this reduction. The data are expressed as the means ± SEM (*n* = 10). ^∗^*P* < 0.05, ^∗∗^*P* < 0.01 vs. NC + saline, ^#^*P* < 0.05 vs. NC + LPS. MWM, Morris water maze; OFT, open field test; NOR, novel object recognition; Lv, lentiviral vectors.

Fourteen days after microinjection of the Lv-*TSPO* or NC vectors, 0.5 mg/kg of LPS or saline was injected intraperitoneally daily. The OFT was conducted before the other behavior tests. Both Lv-*TSPO* and LPS had no effect on line crossing (*P* > 0.05, **Figure 4A**) or rears (*P* > 0.05, **Figure 4B**).

Repeated-measures two-way ANOVA revealed that swimming speed during the 6 days of the MWM was not affected by LPS or Lv-*TSPO* (*F*_treatment_ = 0.4749, *P* = 0.7017; **Figure 4C**). The escape latency improved over time while the acquisition process seemed not to be affected by LPS and Lv-*TSPO* (*F*_time_ = 65.94, *P* < 0.0001, *F*_treatment_ = 2.408, *P* = 0.0687; **Figure 4D**). Interestingly, LPS decreased the crossing times and the time percentage in the target quadrant. Additionally, Lv-*TSPO* reversed the reduction in the probe trial (*F*_LPS_ = 6.23, *P* = 0.0173, *F*_Lv-_*_TSPO_* = 4.225, *P* = 0.0471, *F*_Lv-_*_TSPO_*_×__LPS_ = 3.643, *P* = 0.0643 for crossing time; *F*_LPS_ = 6.952, *P* = 0.0123, *F*_Lv-_*_TSPO_* = 1.961, *P* = 0.1699, *F*_Lv-_*_TSPO_*_×LPS_ = 7, *P* = 0.012 for target quadrant; **Figures 4E,F**). Bonferroni’s multiple comparison showed that NC + LPS mice had less platform crossing times than the NC + saline mice (*P* < 0.01) and Lv-*TSPO* + LPS mice (*P* < 0.05), and they also spent less time in the target quadrant than the NC + saline mice (*P* < 0.01) and Lv-*TSPO* + LPS mice (*P* < 0.05).

In the NOR test, LPS-induced cognitive impairment was shown as the reduction of the recognition object index. Lv-*TSPO* could ameliorate this impairment (*F*_LPS_ = 4.755, *P* = 0.0358, *F*_Lv-_*_TSPO_* = 1.704, *P* = 0.2001, *F*_Lv-_*_TSPO_*_×LPS_ = 7.052, *P* = 0.0117; **Figure 4G**). Our data suggested that the LPS-induced cognitive impairment could be reversed by TSPO overexpression in the dentate gyrus.

### Lv-*TSPO* Suppresses LPS-Induced Microglial Activation and Accumulation of Inflammatory Cytokines in the Hippocampus

**FIGURE 5:**
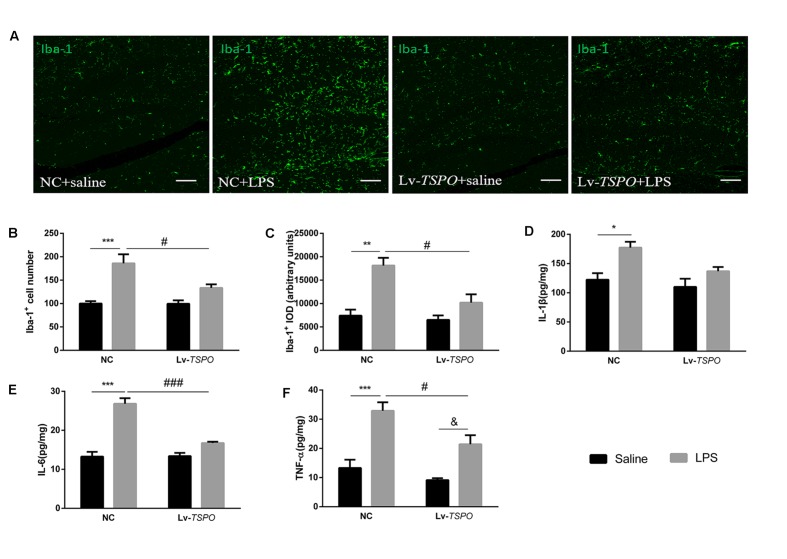
**Lv-*TSPO* suppresses the LPS-induced accumulation of inflammatory cytokines and microglial activation in the hippocampus. (A)** Representative images of Iba1-labeled activated microglia in the hippocampal dentate gyrus. Activated microglia are shown in green. Scale bar = 150 μm. **(B)** Quantification of the Iba1-labeled cells. LPS increased Iba1-labeled cells, while Lv-*TSPO* attenuated this increase. **(C)** LPS increased the integral optical density (IOD) of Iba1 staining, which was attenuated by Lv-*TSPO*. The data are expressed as the means ± SEM (*n* = 4). LPS increased the accumulation of IL-1β **(D)**, IL-6 **(E)** and TNF-α **(F)**, all of which were reversed by Lv-*TSPO*. The data are expressed as the means ± SEM (*n* = 6). ^∗^*P* < 0.05, ^∗∗^*P* < 0.01, ^∗∗∗^*P* < 0.001 vs. NC + saline, ^#^*P* < 0.05, ^###^*P* < 0.001 vs. NC + LPS, ^&^*P* < 0.05 vs. Lv-*TSPO* + saline.

In view of the important roles that microglial activation plays in inflammatory cognitive impairment (Annane and Sharshar, 2015), we investigated the expression of the Iba1 protein in the dentate gyrus (**Figure 5A**). Our results showed that LPS clearly caused microglial activation while Lv-*TSPO* suppressed microglial activation (*F*_LPS_ = 28.30, *P* = 0.0002, *F*_Lv-_*_TSPO_* = 5.520, *P* = 0.0367, *F*_Lv-_*_TSPO_*_×LPS_ = 5.314, *P* = 0.0398 for number; *F*_LPS_ = 24.63, *P* = 0.0003, *F*_Lv-_*_TSPO_* = 9.275, *P* = 0.0102, *F*_Lv-_*_TSPO_*_×LPS_ = 5.911, *P* = 0.0317 for density; **Figures 5B,C**).

We also investigated the levels of several pro-inflammatory cytokines (IL-1β, IL-6 and TNF-α) in the hippocampus. An ELISA assay showed that LPS caused obvious increases in IL-1β (*F*_LPS_ = 14.28, *P* = 0.0012, **Figure 5D**), IL-6 (*F*_LPS_ = 66.04, *P* < 0.0001, **Figure 5E**) and TNF-α (*F*_LPS_ = 38.26, *P* < 0.0001, **Figure 5F**). Lv-*TSPO* reversed the LPS-induced accumulation of pro-inflammatory cytokines (*F*_Lv-_*_TSPO_* = 5.916, *P* = 0.0245, *F*_Lv-_*_TSPO_*_×LPS_ = 1.663, *P* = 0.2119; *F*_Lv-_*_TSPO_* = 22.9, *P* = 0.0001, *F*_Lv-_*_TSPO_*_×LPS_ = 24.19, *P* < 0.0001; *F*_Lv-_*_TSPO_* = 9.186, *P* = 0.0066, *F*_Lv-_*_TSPO_*_×LPS_ = 2.011, *P* = 0.1716; for IL-1β, IL-6, and TNF-α, respectively). These data indicated that TSPO overexpression partially reversed the accumulation of pro-inflammatory cytokines and microglial activation in hippocampus induced by LPS.

### Lv-*TSPO* Ameliorates the Reduction of Neurogenesis Caused by LPS

**FIGURE 6:**
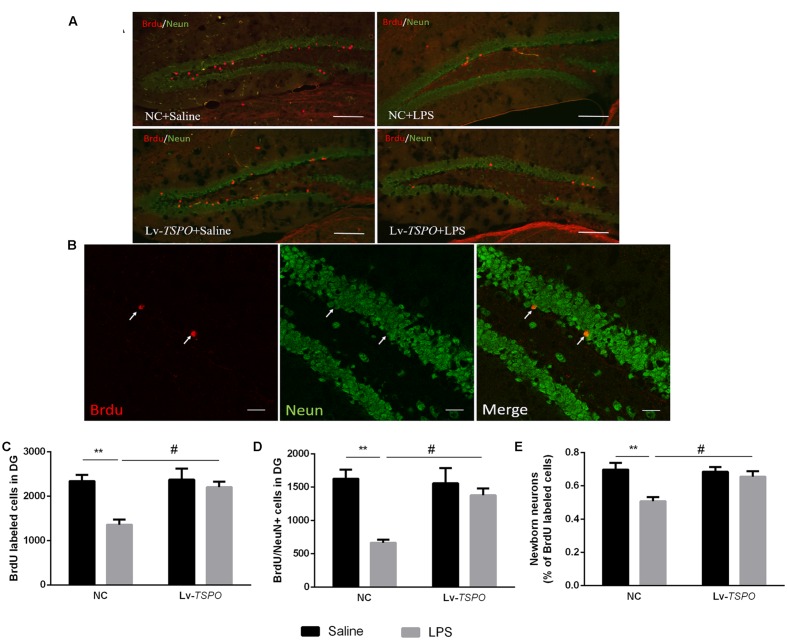
**Lv-*TSPO* ameliorates the reduction of neurogenesis caused by LPS. (A)** Micrographs and quantification of Brdu-labeled cells (red) in the dentate gyrus. Scale bar = 200 μm. **(B)** Confocal micrographs and quantification of NeuN (green) and Brdu (red) co-labeled cells in the dentate gyrus. Scale bar = 50 μm **(C)** Brdu-labeled cells were decreased by LPS, which was reversed by Lv-*TSPO*. **(D)** LPS decreased the number of NeuN- and Brdu- co-labeled cells, which was reversed by Lv-*TSPO*. **(E)** The percentage of new neurons among the Brdu-labeled cells was decreased by LPS, while this was attenuated by Lv-*TSPO*. The data are expressed as the means ± SEM (*n* = 4). ^∗∗^*P* < 0.01 vs. NC + saline, ^#^*P* < 0.05 vs. NC + LPS.

Given that neurogenesis could be reduced by inflammation and that this reduction might be linked with cognitive impairment (Kohman and Rhodes, 2013; Chesnokova et al., 2016), we labeled Brdu- and NeuN-positive cells in the dentate gyrus by immunofluorescent means (**Figures 6A,B**). LPS reduced not only the number of Brdu-positive cells (*F*_LPS_ = 12.22, *P* = 0.0044, **Figure 6C**) but also the number of cells co-labeled with Brdu and NeuN (*F*_LPS_ = 15.66, *P* = 0.0019, **Figure 6D**), as well as the percentage of co-labeled cells of total Brdu-positive cells (*F*_LPS_ = 11.79, *P* = 0.0049, **Figure 6E**). Lv-*TSPO* reversed this reduction at different levels (*F*_Lv-_*_TSPO_* = 7.128, *P* = 0.0204, *F*_Lv-_*_TSPO_*_×LPS_ = 6.056, *P* = 0.03 for Brdu^+^ cells; *F*_Lv-_*_TSPO_* = 4.951, *P* = 0.046, *F*_Lv-_*_TSPO_*_×LPS_ = 7.369, *P* = 0.018 for Brdu^+^/NeuN^+^ cells; *F*_Lv-_*_TSPO_* = 4.406, *P* = 0.0576, *F*_Lv-_*_TSPO_*_×LPS_ = 6.194, *P* = 0.0285, for the percentage of new neurons in Brdu^+^ cells). These data indicate that TSPO overexpression attenuated the impaired hippocampal neurogenesis induced by LPS.

## Discussion

The present study demonstrated that overexpression of TSPO in the hippocampal dentate gyrus significantly suppressed the cognitive impairment induced by LPS in mice and that this effect might be mediated by the attenuation of inflammatory cytokines, inhibition of microglial activation, and promotion of neurogenesis.

Systemic LPS administration can cause blood-brain barrier disruption and neuroinflammation through a series of mechanisms, inducing cognitive decline (Weintraub et al., 2013). However, the method of LPS administration changed from single to consecutive, and the dose applied to induce cognitive dysfunction varied from 0.125 to 1 mg/kg (Wang et al., 2013; An et al., 2015; Sun et al., 2015; Thangarajan et al., 2015). Additionally, LPS-induced low locomotor activity may have had an influence on the behavioral tests. It has also been reported that following seven consecutive days of LPS (0.25 mg/kg) administration, locomotor activity returned to normal levels (Kahn et al., 2012). In the present study, we intended to investigate the cognitive performance of mice beginning 1 day after the first LPS administration. Thus, we optimized the dose and time of LPS administration so as to not affect locomotor activity at first. We chose three doses of LPS (0.2, 0.5, and 1 mg/kg, *i.p.* once a day for 6 days) based on previous reports (Wang et al., 2013; An et al., 2015; Sun et al., 2015; Thangarajan et al., 2015). Our data showed that locomotor activity recovered after 20 h except in the 1 mg/kg mice. Therefore, 0.5 mg/kg LPS (*i.p.* daily) was chosen, and the subsequent behavioral tests were carried out 20 to 24 h after LPS administration.

Translocator protein is expressed widely in different organs of the body, including the testis, the adrenal cortex, ovarian granulose and luteal cells, the placenta and brain glial cells (Papadopoulos et al., 2006). To exclude the systemic effects of TSPO ligands, we overexpressed TSPO in the bilateral hippocampus using a lentivirus, which mediates specific protein overexpression that can last for months (Krassnig et al., 2015; Chen et al., 2016). As a marker, EGFP is often applied to trace the location of lentiviral-mediated protein overexpression (Eerola et al., 2014). In the present study, the specific expression of EGFP (green) was observed in the dentate gyrus after intra-hippocampal injection of the Lv-*TSPO*, indicating that the method of local overexpression was reliable. Western blot also suggested the successful fusion of the TSPO gene by lentiviruses. All of these data suggested that TSPO was highly expressed in the DG of the hippocampus after intra-hippocampal injection of Lv-*TSPO*. As the lentiviral vector stimulated hippocampal TSPO signaling efficiently, we then examined whether this molecular change modified cognitive performance in an LPS-induced cognitive impairment model. Cognitive performance was evaluated by MWM and NOR, two robust and reliable tests that are strongly correlated with hippocampal-dependent memory (Vorhees and Williams, 2006; Li et al., 2011) that are widely used in evaluating cognitive deficits induced by LPS (Vorhees and Williams, 2006; Belarbi et al., 2012; Dong et al., 2013; Fruhauf et al., 2015; Zhang et al., 2015). In our present study, LPS induced clear cognitive deficits, exhibited as a decrease in the recognition index in NOR and a decrease in platform crossing and target quadrant occupancy during the MWM. All of these behavioral changes were reversed by hippocampal TSPO overexpression. Our present results are consistent with other reports that found that TSPO ligands (PK11195) exhibited pro-cognition effects in an LPS-induced cognitive impairment model (Ma et al., 2016), while the possible mechanisms are worthy of further investigation.

In an effort to better understand the anti-cognitive-impairment effect of hippocampal overexpression of TSPO, we then tested the possible mechanisms. Evidence has suggested that the inflammatory process in the CNS is characterized by microglial activation and the accumulation of pro-inflammatory cytokines. In the event of brain insults, such as inflammation and neurodegeneration, microglia activate by undergoing morphological transformation from a “ramified” resting state to an active, motile “amoeboid” state (Fields et al., 2014). It is also reported that microglial activation is closely related to LPS-induced cognitive impairment (Fields et al., 2014). Iba1 is a 17 kDa protein that is specifically expressed in macrophages and microglia. An increase in the IOD for Iba1 staining was interpreted to signify microglial activation. Consistent with previous reports, which revealed that TSPO ligands suppressed microglial activation (Zhao et al., 2011; Bae et al., 2014; Karlstetter et al., 2014). In the present study, TSPO overexpression in the dentate gyrus reversed the number and density of Iba1-labeled cells, indicating that TSPO overexpression suppressed LPS-induced hippocampal microglial activation.

Microglial activation stimulates the accumulation of pro-inflammatory cytokines, such as IL-1β, IL-6, and TNF-α, which are responsible for memory impairment induced by LPS (Van Dam et al., 1995; Smith et al., 2012; Skelly et al., 2013). Previous studies suggested that the overexpression of TSPO ameliorates the LPS-induced accumulation of pro-inflammatory cytokines in BV2 cells, while TSPO knock-down produced the opposite effect (Bae et al., 2014). Moreover, numerous TSPO ligands (XBD173, vinpocetine, RO5-4864) could attenuate microglial activation and the accumulation of pro-inflammatory cytokines *in vitro* and *in vivo* (Zhao et al., 2011; Bae et al., 2014; Karlstetter et al., 2014). Consistent with these findings, we demonstrated that LPS increased the production of IL-1β, IL-6 and TNF-α, which was reversed by TSPO overexpression and is also consistent with behavioral tests.

Studies have demonstrated that adult hippocampal neurogenesis plays a major role in learning and memory, and impairment of hippocampal neurogenesis leads to cognitive deficits (van Praag et al., 2002; Lazarov and Marr, 2010; Ekdahl, 2012). In addition, hippocampal neurogenesis was suppressed by many factors, such as microglial activation and pro-inflammatory cytokines (van Praag et al., 2002; Green et al., 2012; Ekdahl, 2012; Chesnokova et al., 2016). Our present study found that overexpression of TSPO suppressed microglial activation and the accumulation of pro-inflammatory cytokines in the DG of the hippocampus. Allopregnanolone, which is promoted by TSPO overexpression, might modulate neurogenesis in rodents (Brinton and Wang, 2006; Charalampopoulos et al., 2008). Therefore, we hypothesized that Lv-*TSPO* and the subsequent synthesis of allopregnanolone targeting adult neurogenesis may be beneficial for the improvement of cognitive impairment. To address our hypothesis, we next investigated the effect of hippocampal TSPO overexpression on hippocampal neurogenesis. We found that chronic treatment with LPS decreased hippocampal neurogenesis, while hippocampal TSPO overexpression reversed the impaired hippocampal neurogenesis, which was consistent with previous reports that revealed that anti-inflammatory drugs, as well as allopregnanolone, promoted neurogenesis in animal models (Brinton and Wang, 2006; Charalampopoulos et al., 2008; Varnum et al., 2015; Vay et al., 2016). Thus, we considered that the reversal of the impaired neurogenesis induced by LPS contributed to the neuroprotective effects of TSPO overexpression.

## Conclusion

To our knowledge, this is the first report of the mechanisms of lentiviral-mediated TSPO overexpression in the dentate gyrus against neuroinflammatory cognitive impairment. It should also be stated that in the present study, we did not antagonize the effect of allopregnanolone. Therefore, we could not completely attribute the anti-inflammatory effects of TSPO overexpression to allopregnanolone. Nevertheless, the results of these investigations advanced our knowledge of TSPO during neuroinflammatory cognitive dysfunction, which is a potential therapeutic target for the treatment of inflammatory cognitive deficits.

## Author Contributions

WW helped to conceive the study, formulated the design of the study, carried out the execution and acquisition of the data, and drafted the manuscript. LZ and RX helped to analyze and interpret the data. XZ, LL, QF and WZ helped to carry out the execution of the study. WM and YL provided funding for the project, conceived of the study, participated in its design and coordination and helped to draft the manuscript. All authors read and approved the final manuscript.

## Conflict of Interest Statement

The authors declare that the research was conducted in the absence of any commercial or financial relationships that could be construed as a potential conflict of interest.
